# Laser systems for time-resolved experiments at the Pohang Accelerator Laboratory X-ray Free-Electron Laser beamlines^1^

**DOI:** 10.1107/S1600577519003515

**Published:** 2019-04-15

**Authors:** Minseok Kim, Chang-Ki Min, Intae Eom

**Affiliations:** a Pohang Accelerator Laboratory, Pohang, Gyeongbuk 37673, Republic of Korea

**Keywords:** XFELs, time-resolved XFEL experiments, optical lasers, beamlines, amplifier systems, fundamental waves

## Abstract

Optical laser systems for ultrafast X-ray sciences have been established at the Pohang Accelerator Laboratory X-ray Free-Electron Laser (PAL-XFEL) beamlines. Three Ti:sapphire regenerative amplifier systems are synchronized to the XFEL with femtosecond precision, and the low temporal jitter of the PAL-XFEL results in an experimental time resolution below 150 fs (full width at half-maximum). A fundamental wave and its harmonics are currently provided for all beamlines, and tunable sources from ultraviolet to near-infrared are available for one beamline. The position stability of the optical laser extracted from the intensity-based center of mass at the sample position is less than 3% (r.m.s.) of the spot size.

## Abstract 2

The experimental lasers at the PAL-XFEL beamlines, from the source to the sample position, are described.

## 1. Introduction   

The intense, ultrashort and highly coherent pulses from X-ray free-electron lasers (XFELs) have opened new fields of ultrafine and ultrafast X-ray sciences in physics, chemistry and biology (Bergmann *et al.*, 2017 ▸). In particular, direct observation of atomic-scale changes such as the formation/dissociation of chemical bonds (Kim *et al.*, 2015 ▸; Suga *et al.*, 2017 ▸), collective atomic motions in solids (Fritz *et al.*, 2007 ▸; Gerber *et al.*, 2017 ▸) and phase transitions (Gaudin *et al.*, 2012 ▸) has become possible with femtosecond-scale time resolution.

Time-resolved X-ray experiments are typically performed with the pump–probe technique (Minitti *et al.*, 2015 ▸). Any method of initiating dynamic processes in matter may act as a pump (Jo *et al.*, 2011 ▸; Wang *et al.*, 2014 ▸); among which, femtosecond laser pulses are the most accessible tool for making instantaneous changes in materials. In this case, the XFEL probes the photo-induced change caused by the pump pulse after some time-delay, *t*. The laser system and its beam-delivery paths should be located and maintained in a controlled environment for reliable provision of optical pulses in the experiments. In addition, the optical laser and the XFEL must be precisely synchronized to not deteriorate the experimental time resolution. Finally, optical laser pulses tuned to the absorption wavelength of the sample have to be delivered to certain interaction points along the XFEL beamline. In this paper, we report on the optical laser systems for time-resolved XFEL experiments currently proceeding at the Pohang Accelerator Laboratory (PAL) beamlines. We describe the paths from the laser output to each sample position and the available laser parameters as well as the synchronization scheme and experimental conditions in terms of the optical laser.

## 2. Laser facilities at PAL-XFEL beamlines   

Each beamline currently operating at the PAL-XFEL has a dedicated optical laser system for time-resolved XFEL experiments. The laser systems in the beamlines have similar configurations, which consist of a Ti:sapphire oscillator (Vitara-T, Coherent, Inc.) and a regenerative amplifier followed by a single-pass amplifier (Legend Elite DUO HE, Coherent, Inc.). To minimize the instabilities of the system, all components except the pulse compressor are positioned in a cleanroom environment whose temperature, relative humidity on the optical table, and particle density are maintained at 24°C ± 0.5°C, 45% ± 5% and Class 10 000 (ISO 7), respectively.

Amplified, uncompressed laser pulses are delivered from the laser room to the beamline through an evacuated tube. Therefore, the beam path is safely isolated from the outside, and nonlinear effects as well as spectral and temporal dis­tortions caused by high peak power can be suppressed. The intrinsic beam divergence and position instability of the Ti:sapphire laser could lead to poor beam quality and fluctuation at a distant point. Therefore, we employ telecentric relay imaging by using a concave–convex doublet (each formed with a concave and convex lens) at each end of the evacuated beam tube and we employ active beam-pointing stabilization during beam transportation.

A pulse compressor, an optical delay line, and frequency conversion units such as a harmonic generator and an optical parametric amplifier (OPA) are located in a light-tight enclosure at the beamline. Finally, femtosecond laser pulses with appropriate wavelength and intensity excite the sample at the interaction point. Specifications of the laser systems currently operated and the laser parameters at the sample positions are provided in Table 1 ▸.

### 2.1. Optical lasers for hard X-ray beamlines   

The PAL-XFEL operates two hard X-ray (HX) beamlines for X-ray scattering and spectroscopy (XSS) and nano-crystallography and coherent imaging (NCI) research (Ko *et al.*, 2017 ▸; Park *et al.*, 2016 ▸; Park, Kim, Kim *et al.*, 2018 ▸; Kim *et al.*, 2018 ▸). Two identical Ti:sapphire laser systems are installed in the HX laser room located above the NCI hutch structure. This allows the optical laser to go directly down to every laser booth in the hutches, where pulse compression, delay control and frequency conversion take place. In principle, one laser system is assigned per beamline and each laser system can be used as backup for the other if necessary.

The repetition rate of the amplified output can be selected among integer divisions of 120 Hz (Table 1 ▸). During 2018, the PAL-XFEL was operated at a repetition rate of up to 30 Hz, so the most frequently used rates of the optical laser were 15 and 30 Hz for typical optical pump XFEL probe experiments. In the case of the HX optical laser systems, the transform-limited pulse duration is optimized at 100 fs (full width at half-maximum; FWHM).

**Figure 1:**
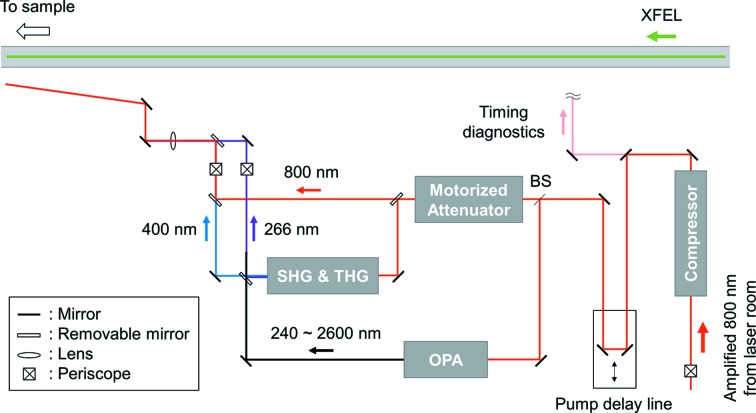
Optical laser layout in the hard X-ray beamlines of the PAL-XFEL.

As shown in Fig. 1 ▸, the laser pulses providing maximum pulse energies of 10 mJ after compression are bifurcated after the delay stage (IMS600LM, Newport). The first optical path is for the 800 nm fundamental and its harmonics (HGS-T, Coherent, Inc.), with pulse energies up to 1 mJ and 0.7 mJ for the second harmonic generation (SHG, 400 nm) and third harmonic generation (THG, 266 nm), respectively. The output pulse energy can be controlled through a motorized attenuator on the 800 nm beam path, consisting of an achromatic λ/2 plate and two reflective thin-film polarizers. The second path is for the OPA system (TOPAS Prime, Light Conversion Ltd) pumped by 3.5 mJ pulse^−1^ centered at 800 nm. The OPA system is capable of providing tunable femtosecond pulses ranging from ultraviolet (>240 nm) to far-infrared (<20 µm). Currently, we provide OPA output up to 2600 nm (only for the XSS beamline) by using a frequency mixer (NirUVis, Light Conversion Ltd) in combination with the OPA system. A difference frequency generation unit (NDFG, Light Conversion Ltd) capable of producing wavelengths up to 20 µm will be added later. Finally, before focusing to the sample position, the beam height is adjusted by a periscope to be equal to that of the XFEL.

For time-resolved serial femtosecond crystallography (SFX) experiments we also provide a nanosecond *Q*-switched laser (Minilite II, Continuum) at the NCI beamline (not shown in Table 1 ▸). The available wavelengths are 1064 nm (<50 mJ pulse^−1^) from the Nd:YAG laser, its harmonics (532 nm and 266 nm) and 355 nm with repetition rates of up to 15 Hz. The energy fluctuation and typical pulse duration of the nanosecond laser are 2.6% (r.m.s.) and 5 ns ± 2 ns, respectively.

### 2.2. Optical lasers for a soft X-ray beamline   

The pump–probe experiments at the soft X-ray (SX) scattering and spectroscopy (SSS) beamline, which includes the X-ray absorption spectroscopy/X-ray emission spectroscopy (XAS/XES) station (Park, Kim, Min *et al.*, 2018 ▸) and the resonant SX scattering (RSXS) station, are supported by a 4 mJ Ti:sapphire laser system installed in the laser room located at the most downstream end of the SX experimental hall. After the external pulse compressor at the experimental hall, the optical laser provides a transform-limited pulse duration of 40 fs (FWHM) operated up to 1.08 kHz. Specifically, the repetition rate of the optical laser can be selected among integer divisions of 1.08 kHz through control of the pulse slicer outside the regen cavity.

**Figure 2:**
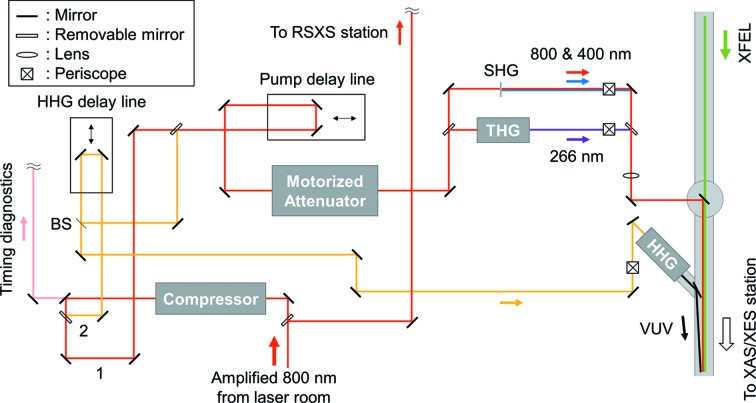
Optical laser layout in the soft X-ray XAS/XES end-station of the PAL-XFEL.

Fig. 2 ▸ depicts the optical layouts of the XAS/XES end-station in the SX beamline. After the external pulse compressor, two optical paths can be selected via removable mirrors. The first optical path is used for delivering typical pump sources (*i.e.* 800 nm, 400 nm and 266 nm) through a laser-in-couple chamber. The pump intensity is adjusted with a Watt Pilot motorized attenuator (Altechna). The pump delay line (IMS600LM, Newport) has a travel range of 600 mm, which corresponds to a time delay of 4 ns. A second path (under development) is planned for a vacuum ultraviolet (VUV) source in the range 20–100 eV based on the high harmonic generation (HHG) process (Park, Kim, Min *et al.*, 2018 ▸). A beam splitter after the HHG delay line (ILS300LM, Newport) equally bifurcates 800 nm pulses so that one arm can share the path for the pump beam including the pump delay line. Finally, at the XAS/XES end-station, various combinations of two-color time-resolved experiments will be possible by utilizing SX FEL, optical laser and VUV pulses. Regardless of optical path, before the laser-in-couple chamber (or the end-stations), the beam height is adjusted by a periscope to be equal to that of the XFEL. Meanwhile, the RSXS end-station is in preparation at the SSS beamline and the optical laser setup will be integrated accordingly.

### 2.3. Synchronization with XFEL   

**Figure 3:**
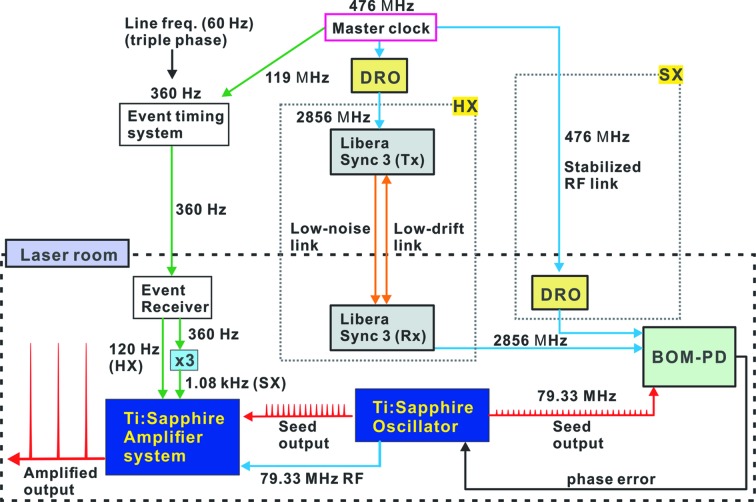
Synchronization map between the experimental laser systems and the PAL-XFEL timing system. DRO: dielectric resonator oscillator; BOM-PD: balanced optical and microwave phase detector.

To achieve the highest experimental time resolution, the relative phase between the XFEL and the optical laser should be fixed as precisely as possible. The operation scheme is shown in Fig. 3 ▸. All laser systems in the PAL-XFEL beamlines are synchronized with an S-band radiofrequency (RF) clock of 2856 MHz and the event-timing system phase-locked to the master clock of 476 MHz with a line frequency of 60 Hz. Because the event timing is related to the XFEL beam rate, one of the frequencies from the event receiver acts as an external trigger for the laser amplifier. The HX laser systems use 120 Hz, whereas the SX laser system uses 1.08 kHz, *i.e.* triple the 360 Hz fundamental event clock.

The S-band RF is used as a reference for cavity-length feedback, allowing the oscillator to operate at 79.33 MHz, *i.e.* 1/36 of the RF (Min *et al.*, 2016 ▸). For the HX beamlines, the RF (2856 MHz) is directly distributed through a commercial reference clock transfer system (Libera Sync 3, Instrumen­tation technology) with two single-mode fibers, which compensate for phase drift caused by environmental changes (Zorzut *et al.*, 2015 ▸). In contrast, the SX laser system currently uses the reference 2856 MHz from a dielectric resonator oscillator (GPLDRO2856, Ingenieurbüro Gronefeld) externally locked to the RF timing link at 476 MHz. In this case, the low-loss cables used for the 476 MHz link (SUCOFEED_1_5/8_LA, Huber+Suhner) show temperature-dependent phase drift at 130 fs m^−1^ K. Additionally, we measured the daily temperature variation of the RF link to be 0.08°C (peak-to-peak) in the 100 m-long SX undulator section, whereas it was measured to be 0.01°C (peak-to-peak) in the 90 m-long SX experimental hall. Overall, ∼1 ps of phase drift has been observed over 10 h during experiments (data not shown). For the SX beamline, we plan to implement the same synchro­nization scheme as used in the HX beamlines.

A balanced optical and microwave phase detector (BOM-PD) built in-house based on a Sagnac interferometer is used for the cavity-length feedback of the Ti:sapphire oscillators (Kim *et al.*, 2006 ▸). It compares the phase error between the RF reference and the oscillator output and controls the cavity length to minimize the error signal. The residual phase jitter from 1 Hz to 100 kHz was 14 fs as measured by the out-of-loop method (Min *et al.*, 2016 ▸).

## 3. Time-resolved XFEL experiments with femtosecond optical laser pulses   

### 3.1. Stability of the optical laser   

**Figure 4:**
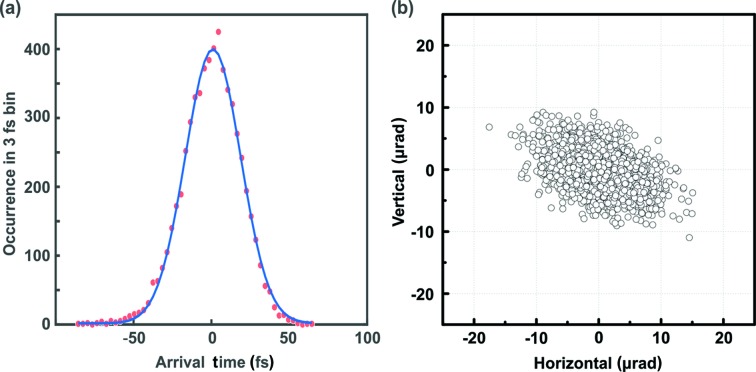
(*a*) Histogram of the arrival-time jitter for 6000 XFEL shots and (*b*) position stability for 1 h of optical laser (800 nm) measured at the XSS beamline. Width of the timing jitter was 42 fs (FWHM) and the position jitter (intensity-based center of mass) was less than 3% (r.m.s.) of the beam diameter. In the position measurement, a 250 µm (1/*e* ^2^ width) focal spot was formed by the 1.5 m focal length lens.

Although the optical laser and the XFEL are synchronized within femtosecond­-level precision, temporal jitter always exists between them at the sample position in the beamline because the optical paths and noise sources differ. The arrival time of the XFEL relative to the optical laser was measured at the XSS beamline using the spectral encoding method (Harmand *et al.*, 2013 ▸; Bionta *et al.*, 2014 ▸). Briefly, a 2 µm Si_3_N_4_ membrane was pumped by the XFEL pulse with a flux density of 370 mJ cm^−2^ at 7.0 keV and probed with a white light con­tinuum generated by 800 nm, 100 fs optical pulses. The induced trans­mission change was recorded by a spectroscopic charge-coupled device (iVAC 324 FI, Andor Technology) after a spectrograph (SP-2300i, Prince­ton Instruments) with 500 nm blazed grating at 300 lines mm^−1^. Fig. 4 ▸(*a*) presents the timing-jitter statistics for the 6000 XFEL shots, for which the measured width of the jitter was 42 fs (FWHM). The instrument response function extracted from the time-resolved diffraction measurement for thin-film Bi(111) was 137 fs (FWHM) without jitter correction (Kang *et al.*, 2017 ▸), which has good correspondence with the estimated value based on the pulse durations, arrival-time jitter and geometrical factor.

The position stability of the optical laser is another key factor in pump–probe experiments. The beam position at the sample is easily affected by environmental changes, as the optical laser in the beamline passes through numerous optics along the optical path, which is at least 15 m at this facility. Moreover, a slightly misaligned beam path or wobble motion in the mechanical delay stage may lead to considerable drift of the beam position. We further minimized the drift by adding the beam stabilization loop (Compact, MRC systems GmbH) after the delay line.

The position stability was measured for 1 h at the sample position of the XSS beamline. A focal spot formed by the *f* = 1.5 m lens was imaged with a long-distance microscope and a camera. The imaging system was calibrated by using a 300 µm pinhole. No notable variation of the spatial beam profile was observed during the measurement. Finally, the beam position was extracted from the intensity-based center of mass of the profile. The angular stability [Fig. 4 ▸(*b*)] at the sample position was 4.7 µrad (H) and 3.2 µrad (V) in r.m.s., which corresponds to less than 3% of the 250 µm (1/*e* ^2^ width) spot size.

### 3.2. Overlap with XFEL   

Prior to the pump–probe experiments, the optical laser and the XFEL must form both temporal and spatial overlaps at a given point of the sample. The pump–probe pulses are simultaneously detected by a fast GaAs photodiode (G4176, Hamamatsu) with a rise time of 30 ps. While monitoring with a 12.5 GHz oscilloscope (DPO71254C, Tektronix), the coarse temporal overlap can be set through a time-delay stage after the phase-locking of the BOM-PD. To reduce ambiguity, this process is repeated whenever the wavelength is changed. Once the spatial overlap is set at the interaction point, the time-zero position can be found by scanning transient responses from reference materials, *e.g.* Bi(111), YAG or Si_3_N_4_. In the case of liquid-phase samples, a change of the wide-angle X-ray scattering signal from the solvent by the optical pump is often useful. Finally, the spatial overlap can be optimized further by monitoring the transient-signal amplitude in real time or by comparing a damaged spot on a wafer at the sample plane.

## 4. Conclusion   

During the second half of 2018, the optical pump laser became accessible for all time-resolved experiments at the PAL-XFEL beamlines. In addition to the laser wavelengths currently provided, we aim to extend the available wavelength to far-infrared and further to terahertz radiation at the XSS beamline, and UV to near-infrared OPAs will also be added to the NCI and SSS beamlines. Thanks to highly accurate synchronization and low timing jitter between the XFEL and the optical laser, experimental time resolutions of less than 150 fs have been achieved without jitter correction. Additional beam-position feedback before the sample made it possible to maintain spatial overlap without considerable position drift over the user beam time. We plan to apply feedback devices to all beamlines of the PAL-XFEL. Finally, we are going to introduce a high-resolution RF phase shifter for the laser oscillator, with which we expect to be able to correct for the slow time drift that could occur during an experiment through an additional delay control in combination with arrival-timing diagnostics.

## Figures and Tables

**Table 1:** Specifications of the Ti:sapphire laser system at the beamlines and their available laser parameters

Experimental hall	HX		SX
Beamline	XSS	NCI	SSS
Repetition rate (MHz) (oscillator)	79.33		
Repetition rate (Hz) (amplifier)	<120		<1080
Fundamental wavelength (nm)	800		
Pulse duration (fs) (FWHM)	100		40
Maximum pulse energy at sample position	5 mJ at 800 nm		4 mJ at 800 nm
	1 mJ at 400 nm		1 mJ at 400 nm
	0.7 mJ at 266 nm		0.5 mJ at 266 nm
Optical parametric amplifier	240–2600 nm	None	
Incident angle to the XFEL	Non-collinear (∼10°)	CXI†: non-collinear (∼15°)	Collinear (∼1°)
	Collinear (∼1°)	SFX: perpendicular (∼90°)	

† CXI: coherent X-ray imaging.
